# Transcanal Endoscopic Repair of Cerebrospinal Fluid Otorrhea Secondary to Congenital Inner Ear Anomaly Using Topical Fluorescein

**DOI:** 10.7759/cureus.78738

**Published:** 2025-02-08

**Authors:** Malak Almalki, Yazeed Aloqailiy, Jihad L Nassar, Renad Alfirm

**Affiliations:** 1 Otolaryngology and Head and Neck Surgery, King Abdulaziz Medical City, Riyadh, SAU; 2 Otolaryngology and Head and Neck Surgery, King Saud University, Riyadh, SAU

**Keywords:** complications of meningitis, duane syndome type 1, endoscopic csf leak repair, inner ear malformation, otitis media with of effusion

## Abstract

An eight-year-old female, known to have sensorineural hearing loss due to inner ear anomalies, presented with a history of recurrent attacks of right acute otitis media complicated by bacterial meningitis. Temporal bone computed tomography showed right middle ear and mastoid effusion without bony dehiscence or erosion, with features of common cavity inner ear anomaly on the right side. Given the patient’s inner ear malformation, cerebrospinal fluid (CSF) otorrhea was suspected. Further evaluation with myringocentesis and beta trace protein analysis confirmed the diagnosis. The patient underwent transcanal endoscopic right ear exploration. Initially, the site of the defect was not revealed; the CSF leak was successfully localized at the oval window, surrounding the stapes footplate. This is the first report, to the best of our knowledge, describing topical fluorescein application in a transcanal endoscopic approach to obliterating CSF otorrhea due to inner ear malformation.

## Introduction

Liquid cerumen, otitis media, and otitis externa are considered the most common causes of otorrhea in children [1,2]. Infrequently, cerebrospinal fluid (CSF) leaks can be the cause of otorrhea. CSF leak is a very serious and fatal condition if not treated adequately and correctly, as it can lead to life-threatening conditions such as meningitis. Patients with CSF leak typically present with recurrent episodes of meningitis rather than a single occurrence [3]. Furthermore, CSF leak is most commonly observed after temporal bone trauma, chronic ear disease, ear surgery, or in cases of inner ear malformation [4].

Proper investigation is a must for early detection and proper treatment; this includes clinical history, physical examination, imaging, and audio tests [1]. The standard management of CSF leak due to inner ear malformation is surgery. Multiple approaches have been described, including the middle cranial fossa approach, the transmastoid approach, and the translabyrinthine approach [5-7]. More recently, the transcanal endoscopic approach has been proposed as a minimally invasive approach with good outcomes in most patients [1,3]. Intraoperative CSF leakage localization can be challenging; topical fluorescein has been reported to be safe and sensitive in CSF rhinorrhea [8,9]. This is the first report, to the best of our knowledge, where topical fluorescein was used in a transcanal endoscopic approach to obliterating the inner ear congenital CSF otorrhea.

## Case presentation

**Figure 1:**
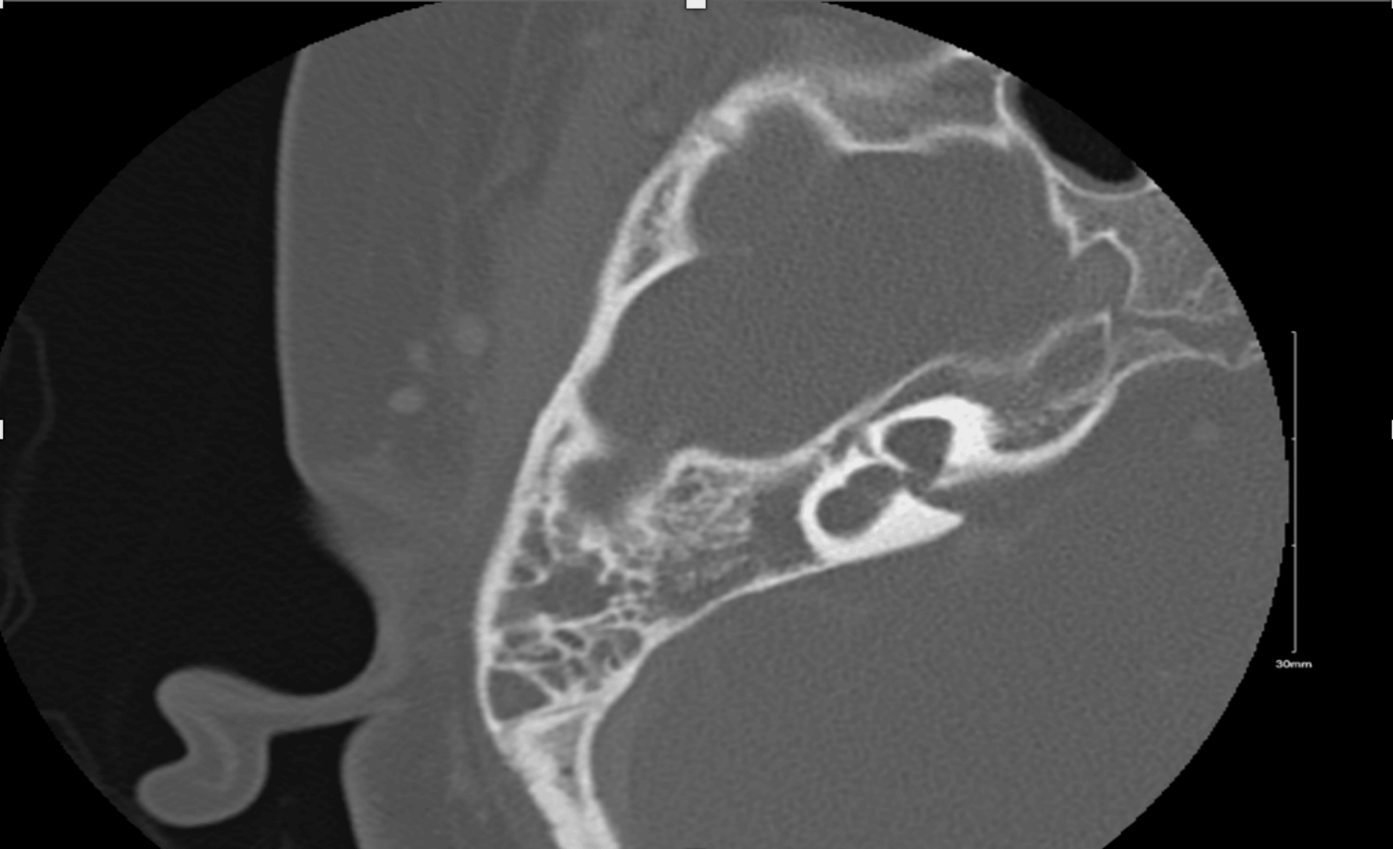
CT shows inner ear malformation with cystic dilatation of the cochlea and vestibule and absence of the semicircular canal. There is also a middle ear and mastoid effusion. The vestibular aqueduct and internal auditory canal are normal

An eight-year-old girl with Duane syndrome type 1, severe sensorineural hearing loss in the right ear, and moderate sensorineural hearing loss in the left ear, managed with a hearing aid. She has common cavity inner ear malformation in the right ear and incomplete partitioning type II on the left side. The patient presented to our hospital with a history of right-sided acute otitis media complicated by bacterial meningitis, which was successfully treated with a full course of intravenous vancomycin and ceftriaxone. A few months later, the patient again developed right-sided acute otitis media, complicated by bacterial meningitis. As the patient was known to have inner ear malformation, suspicion of CSF otorrhea was raised. A temporal bone computed tomography (CT) was performed, revealing the right middle ear and mastoid effusion without evidence of bony dehiscence or erosion, along with features of a common cavity inner ear anomaly on the right side (Figure 1). The patient underwent myringocentesis in the operating room to obtain a fluid sample for further analysis. The sample collected was sent for beta-trace protein (BTP) analysis, which came back confirming the diagnosis of CSF leak. After the complete resolution of meningitis, the patient was taken for transcanal endoscopic right ear exploration.

Surgical technique

**Figure 2:**
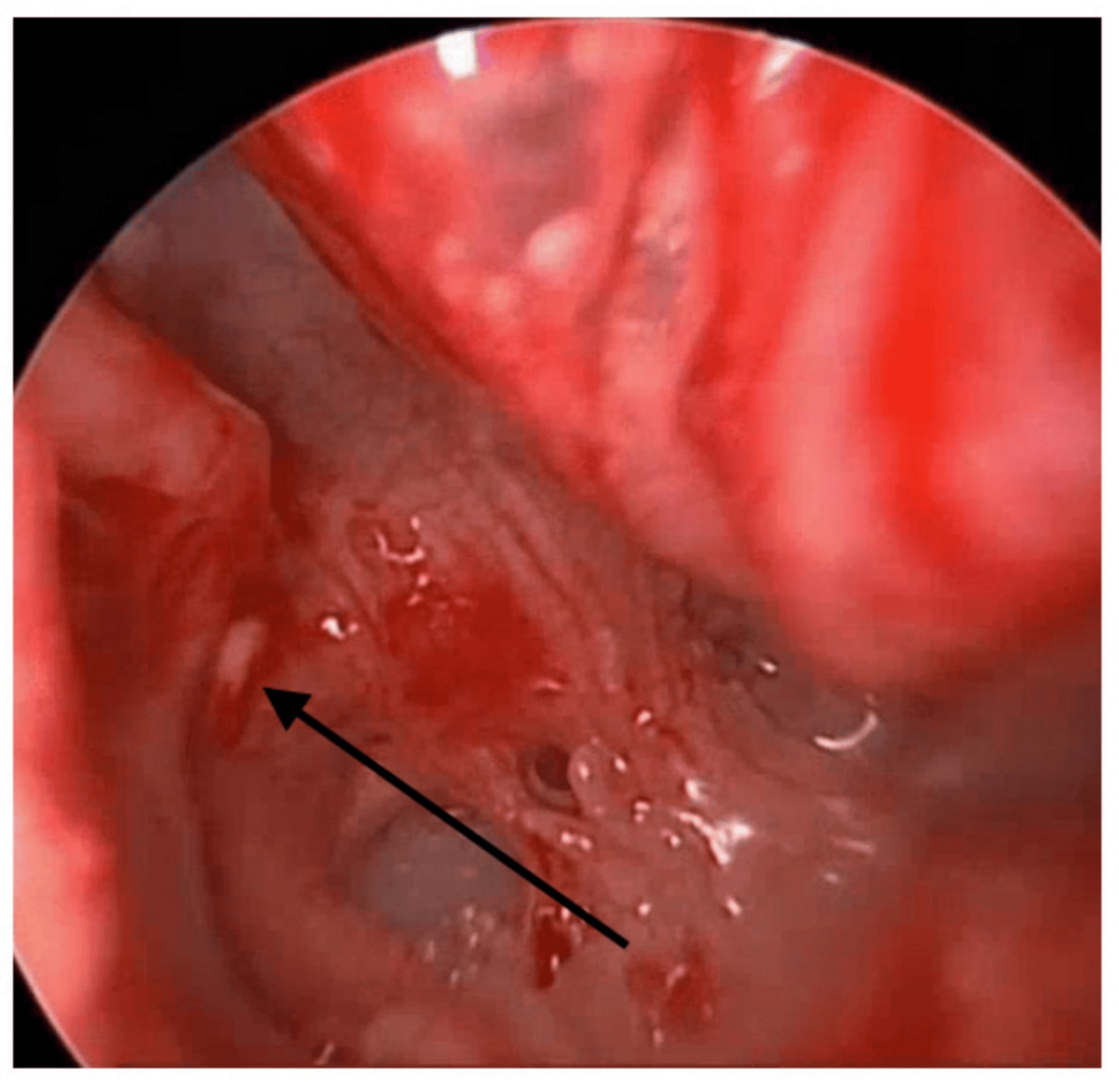
Yellow tissue surrounding the stapes supra-structure (arrow)

**Figure 3:**
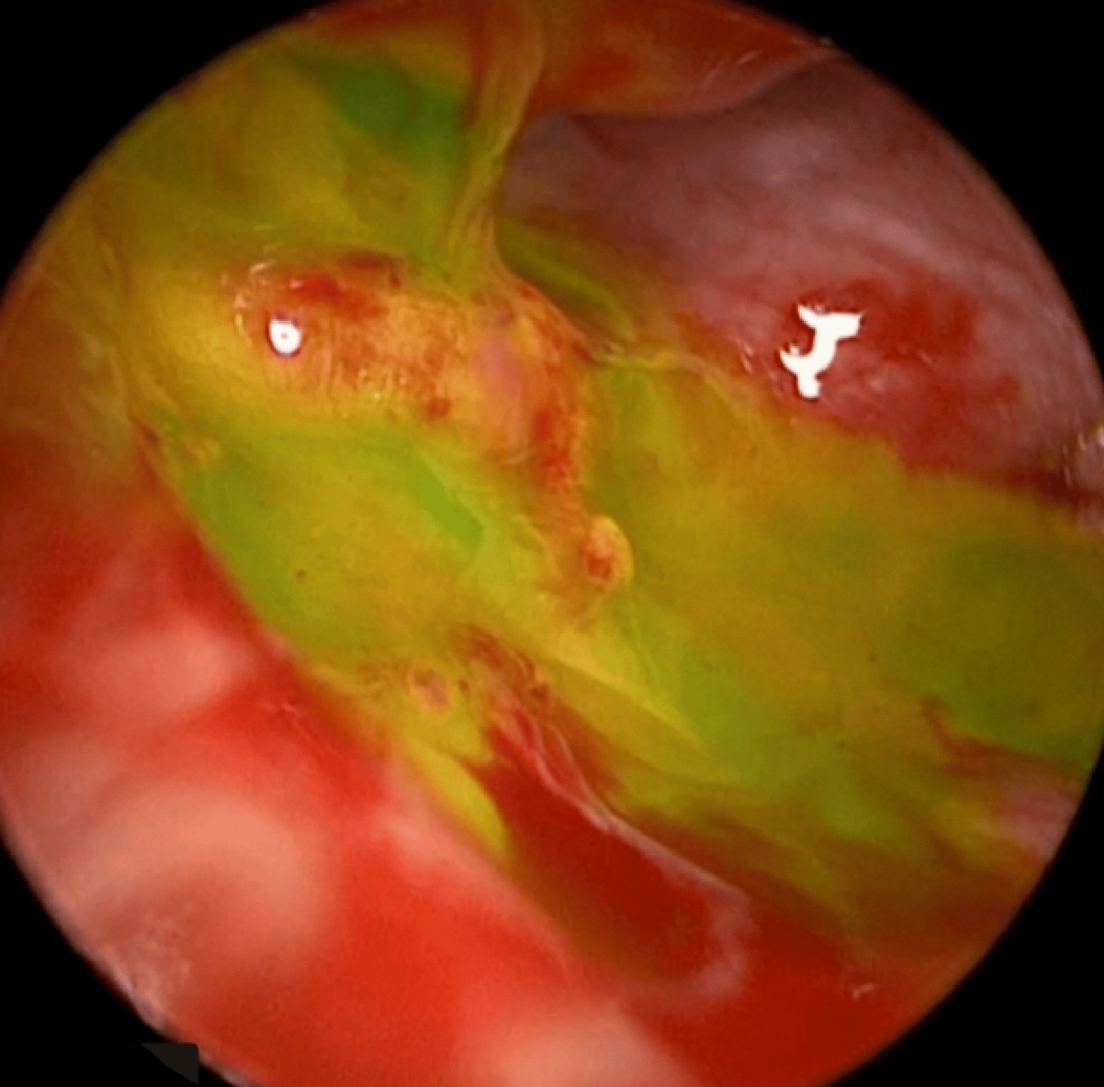
Fluorescein dye was applied in the middle ear, which revealed the CSF leak coming through the oval window, surrounding the footplate of the stapes CSF: cerebrospinal fluid

**Figure 4:**
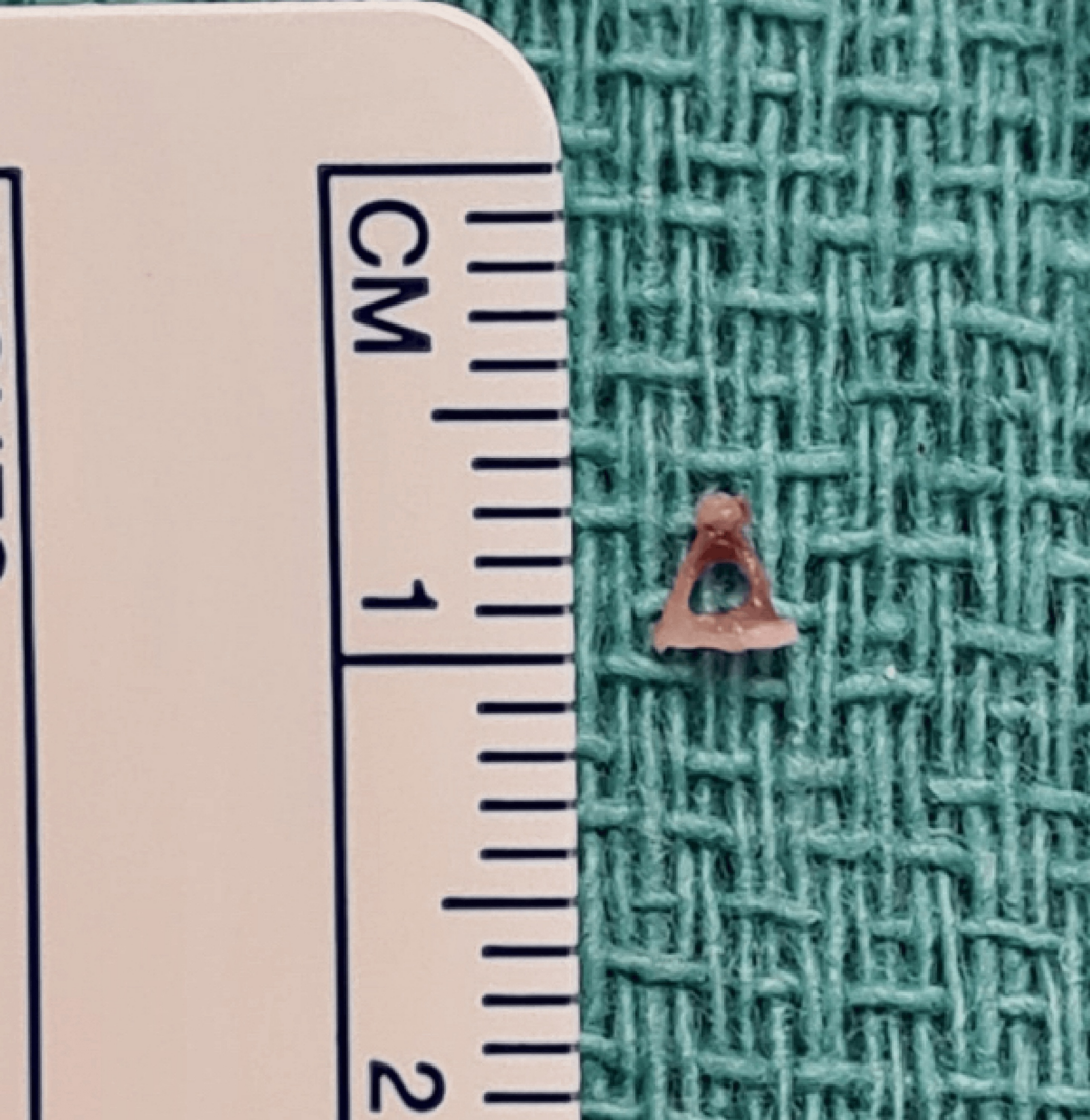
The stapes normal in shape and size

A 0-degree rigid Hopkins rod endoscope (2.9 mm in diameter, 18 cm in length) was used for assessment, revealing a clear external auditory canal and a slightly dull tympanic membrane. Lidocaine (1:100,000) with epinephrine was injected into the external auditory canal and postauricular area. A right facial nerve monitor was connected and continuously used throughout the procedure. The tympanomeatal flap was raised from 6 to 12 o'clock, and the middle ear entry was done. The chorda tympani nerve was identified, and it was preserved throughout the procedure. Atticotomy was done for better visualization of the entire middle ear structure. Initially, there was no middle ear effusion seen. Then assessment of the ossicular chain revealed yellow tissue surrounding the stapes supra-structure (Figure 2); however, the site of the defect was not clear. To aid in localizing the leak, the Valsalva maneuver was performed, and fluorescein dye was applied in the middle ear, which revealed the CSF leak coming through the oval window, surrounding the footplate of the stapes (Figure 3). The incudostapedial joint was separated, and the incus was harvested and saved. Then the temporalis fascia and the muscle were harvested. Removal of the stapes along with the footplate was done as a whole, which revealed a gusher from the oval window. The stapes were normal in shape and size (Figure 4). The harvested fascia and muscle were used to pack the oval window. The previously harvested incus was used to plug the eustachian tube along with extra muscle and fascia for support. A round window was also plugged using harvested fascia and muscle. Fibrin glue (Tisseel) was used to surround the oval window. The facial nerve and chorda tympani were intact throughout the procedure. Valsalva again was done, which revealed no leak. The tympanomeatal flap was tucked down, and it was intact with no perforation. Gelfoam was put in the external auditory canal along with Fucidin ointment and a cotton ball.

Outcome

The patient was observed for more than one year. She was seen on a regular basis in our clinic with no sign of recurrence and no other episode of meningitis. The other ear was observed as well, with no sign of CSF leak.

## Discussion

About 20% of people with congenital sensorineural hearing impairment have a radiologically confirmed inner ear anomaly [10]. Those with inner ear abnormalities are more likely to develop meningitis because of the higher likelihood of improper communication between the subarachnoid and middle ear space. If not properly and effectively managed, CSF leak is highly dangerous, as it can result in permanent neurological sequelae.

Since most patients with inner ear anomalies have normal tympanic membranes, patients with a CSF leak are frequently incorrectly diagnosed as having chronic serous otitis media [11].

Malformations known as incomplete partition I (IP1) are caused by a developmental arrest in the fifth week of pregnancy [12]. A dilated vestibule, a cystic, empty, and unpartitioned cochlea, as well as the lack of a dilated vestibular aqueduct, are the characteristics of an IP1 malformation. A defective fundus that opens into the otic capsule and a dilated internal auditory canal are also features of the majority of IP1 abnormalities [12]. Another common malformation is a common cavity, where the cystic cavity represents the cochlea and vestibule but does not show any differentiation between the cochlea and vestibule. As reviewed in the literature, the oval window accounted for 60% of the fistula location, while the circular window accounted for 13%. The remaining fistula sites included numerous origins, invariably involving the oval window, the hypotympanum, the lower vestibule, the geniculate ganglion, the promontory, and the persisting Hyrtl fissure. According to the research that is currently accessible, the leaks that were discovered in our patient were caused by a stapes footplate problem [3].

Early diagnosis of congenital inner ear dysplasia with CSF otorrhea is very critical and requires a combination of a detailed medical history, audiological testing, and imaging. High-resolution CT can identify CSF leaks as a soft tissue shadow and can even identify the emergence of a liquid level. Magnetic resonance imaging (MRI) can provide an additional supportive tool for diagnosing CSF leakage, and magnetic resonance hydrography (MRH) can show the connected signals of CSF between the vestibule and the internal auditory meatus or tympanic cavity [5,13]. 

Surgical obliteration is the ministry of treatment in patients with inner ear anomalies and CSF otorrhea. Several surgical approaches have been described, including the oval window approach, transmastoid, translabyrinthine, middle fossa, or a combination of these methods. The middle ear or inner ear obliteration can be used to seal off the leakage of inner ear abnormalities. A more recent minimally invasive procedure called transcanal endoscopic ear surgery (EES) uses the external auditory canal rather than a postauricular incision to treat middle ear conditions like tympanic membrane perforation, cholesteatoma, and ossicular fixation/discontinuity. EES has been utilized successfully to treat a variety of lesions that are more medially positioned in the temporal bone, including intralabyrinthine schwannomas, cholesteatomas, and petrous apex cholesterol granulomas [14]. A large field of vision, enhanced illumination, and magnification, as well as less postoperative pain, are all distinct benefits of EES. The endoscope offers the best possible view of the areas of the temporal bone that have previously been difficult to access. The endoscope limitations include the loss of binocular vision, which can be partially made up for by the continuous movement of the endoscope (parallax), the one-handed method, and the requirement for a largely bloodless area [15]. A series of 13 patients with a high success rate has previously detailed the use of transcanal endoscopes to treat CSF leaks caused by inner ear malformation [3]. The drawback is that it might not be able to identify and fix flaws that are hidden from view. An open technique, such as transmastoid or translabyrinthine, may be required if transcanal endoscopes are unable to identify the leakage site.

Several diagnostic modalities are used to detect the site of CSF leakage. Topical fluorescein was described in many cases addressing CSF rhinorrhea. One study reported that topical intranasal administration of 5% fluorescein yielded 100% accuracy in preoperative diagnosis and intraoperative demonstration of the CSF rhinorrhea leak site [8]. This is the first report, to the best of our knowledge, where topical fluorescein was used in the transcanal endoscopic approach to localize the site of congenital CSF otorrhea.

Meningitis risk elimination and maximizing residual hearing are the main therapeutic objectives in the conclusion. Any fistula between the subarachnoid space and the middle ear must be removed to reduce the risk of meningitis. Cochlear implantation is a possibility in patients who still have their cochlear nerves intact. This helps optimize sound perception while also closing the fistula to reduce the chance of contracting meningitis [16]. However, in our case, the patient had an acceptable hearing level on the left side, with good compliance on the hearing aid.

## Conclusions

In conclusion, CSF otorrhea from a congenital inner ear anomaly often presents as persistent clear otorrhea after tympanostomy tube placement or as recurrent meningitis. A minimally invasive transcanal endoscopic approach is a viable alternative to manage this unique entity. Topical fluorescein is an effective, safe tool in guiding endoscopic CSF leak closure.
